# Pathogens—The Hidden Face of Forest Invasions by Wood-Boring Insect Pests

**DOI:** 10.3389/fpls.2019.00090

**Published:** 2019-02-11

**Authors:** Riikka Linnakoski, Kristian M. Forbes

**Affiliations:** ^1^Natural Resources Institute Finland (Luke), Helsinki, Finland; ^2^Department of Virology, University of Helsinki, Helsinki, Finland

**Keywords:** co-infection, disease, forest health, fungi, pathogen, insect pest, invasive species

## Introduction

Forests are one of the most important global vegetation types, serving functions from supporting healthy watershed, to wildlife habitat, and economic industries through their harvesting and tourism value. Understanding and mitigating threats to these resources is therefore of great societal importance. Forest pests are among the most pertinent and obvious threats to forest health, with their impacts amplified by contemporary issues such as climate change and global trade (Allen et al., 2010; Wingfield et al., 2016). In particular, recent increases in the frequency and severity of insect outbreaks have led to the loss of significant forest areas from across the globe (Roy et al., 2014) and highlighted the need for rigorous research to understand the underlying basis of their impacts.

Introduced wood-boring beetles have been particularly damaging, which are often released from competitors and predators in their new environments, and target healthy trees without evolved resistance. In their native range, they typically colonize only dead or dying trees and are, therefore, not recognized as harmful. Recent examples of invasive pests include the emerald ash borer (*Agrilus planipennis*), which primarily attacks ash trees, the redbay ambrosia beetle (*Xyleborus glabratus*) infesting members of the laurel family (*Lauraceae*), and the Asian longhorned beetle (*Anoplophora glabripennis*) and polyphagous shot hole borer (PSHB), which are capable of infesting a wide range of tree species (Baranchikov et al., 2008; Fraedrich et al., 2008; Haack et al., 2010; Eskalen et al., 2013; Paap et al., 2018). Since their accidental introductions from Asia, these aggressive pests have been implicated in the mortality of millions of trees in Europe and North America, and the potential for the further spread of these and other pest species poses a major threat to the health of global forests.

While forest insect pests have understandably attracted strong research attention, especially in countries where forestry is a major economic industry, far less understood are the pathogens they carry and how these contribute to forest damage, especially under climate change. For instance, the redbay ambrosia beetle, PSHB and longhorned beetles described above have each been found to carry assemblages of fungi, including pathogenic species (Fraedrich et al., 2008; Linnakoski et al., 2018; Paap et al., 2018). Here we focus on fungal pathogens vectored by wood-boring insect pests, and argue that they can amplify the negative effects of these pests and cause significant forest damage in their own right (Fraedrich et al., 2008; Ploetz et al., 2013). The purpose of this opinion article is to shed light on these implications, discuss the mechanisms underlying their interactions with host trees, and highlight the research required to resolve gaps in knowledge and progress understanding of this topic.

## Wood-Boring Insects as Fungal Vectors

**Figure 1:**
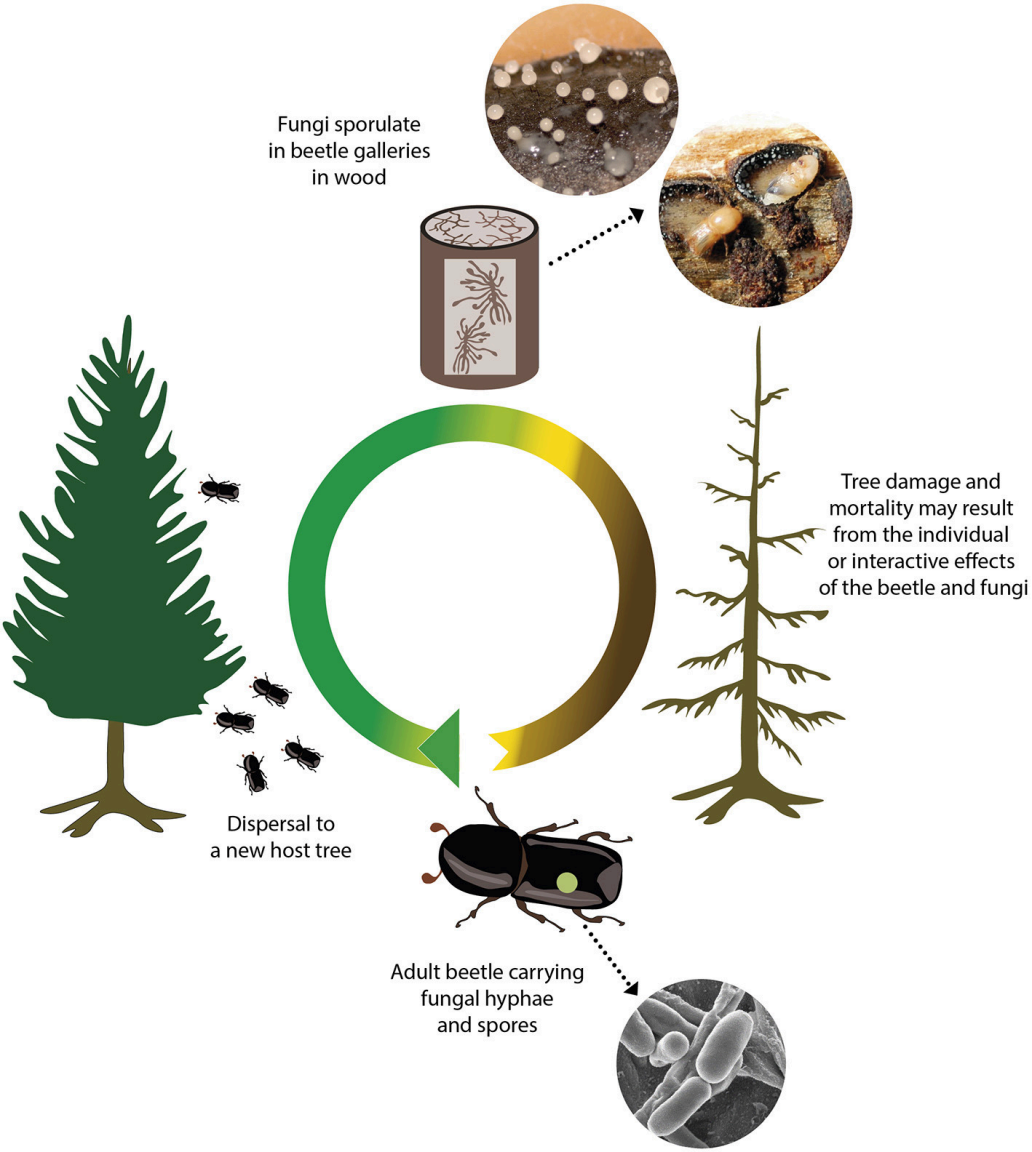
The beetle lifecycle and role in fungi transmission[-2mm][4mm] Q4. Tree damage caused by fungi associated with wood-boring insects is inherently linked to the beetle lifecycle. The beetle life cycle begins following hatching from an egg in a maternal gallery (tunnel) under the tree bark. The larvae remains in the tunnels, feeds on the phloem and gnaws tunnels. During this stage, fungi grow and sporulate and can serve as a source of nutrition for beetle larvae. Larvae pupate and develop into adults under the bark, before flying in search of new host trees and transporting fungi with them. Fungal spores carried on their exoskeleton, in specialized structures called mycangia or via other associated organisms, are then inoculated into a new host tree.

Interactions between fungi and wood-boring bark and ambrosia beetles (Figure 1) are the most intensively studied pathogen-insect relationships in forest ecosystems. The natures of these interactions are diverse, ranging from incidental associations in shared habitats to co-evolved obligate nutritional mutualisms (Farrell et al., 2001; Roe et al., 2011; Hulcr and Stelinski, 2017). Fungal partners benefit from these associations through enhanced transmission, via transport to new trees and habitats.

An increase in the global trade of wood products has enhanced the risk of exotic insect and fungi introductions (Sikes et al., 2018), which may be present in poorly treated timber and wood packaging materials. Scolytine beetles are the most common group of invasive insects detected at border inspections (Lee et al., 2007; Lawson et al., 2018). Their niches overlap with native forest insects in many regions, and as a result, novel insect-fungal interactions are likely to arise. Under this scenario, introduced invasive pathogens may be passed to native insect species to vector, and invasive insect pests may become vectors for native or already established invasive pathogens (Haack, 2006; Wingfield et al., 2016). In both cases, novel fungi-host tree interactions are likely to arise, with unpredictable implications, and indeed, such relationships are increasingly recognized as a concern for forest ecosystems (Lu et al., 2011; Ploetz et al., 2013; Wingfield et al., 2016). Less well acknowledged is the possibility that the same type of novel interactions could result from distributional changes of endemic insect and tree species under conditions of climate change.

Novel beetle-fungus interactions include some of the most important invasive species affecting forest ecosystems, such as the Dutch elm disease and beech bark disease (Ploetz et al., 2013; Santini and Faccoli, 2013; Cale et al., 2017). However, an understanding of the associated fungal assemblages such as these is rare. Indeed, novel and unexpected vectors and host trees have even recently been detected for the extensively studied Dutch elm disease (Jankowiak et al., in press). Most insect and fungal species are not recognized as harmful in their native ranges and have therefore received little research attention. Such fundamental research is further complicated by the need for accurate identification of insects and fungi—these are two of the most species rich groups of organisms, the majority of which are undescribed (Stork et al., 2015; Hawksworth and Lücking, 2017)—but aided by the continual efficacy and affordability of genotyping methods. Establishing baseline information on insect-fungal associations is important for understanding pathogenic potential when beetles expand into new areas, and a lack of such information could hinder the timeliness of risk assessments and mitigation strategies following insect pest range expansions.

## Tree Damage

Tree damage by fungi is inherently linked to the beetle lifecycle (Raffa et al., 2015; Hulcr and Stelinski, 2017). Beetles play important roles in natural (undisturbed by human activity) forest ecosystems, where they typically infest dead or weakened trees and thus participate in forest succession/renewal through the breakdown of biomatter (Raffa et al., 2015). However, occasionally beetles aggregate, especially after natural disturbance events, and mass attack healthy trees in densities sufficient to cause significant damage (Raffa et al., 2015). As part of this process, fungal spores carried on the beetle exoskeleton, in specialized structures called mycangia or via other associated organisms (such as phoretic mites), are inoculated into the tree sapwood (Linnakoski et al., 2016; Hulcr and Stelinski, 2017).

It is not well-known why some beetles that usually colonize dead or stressed hosts in their native range attack healthy trees when arriving in a new area (Hulcr and Dunn, 2011). Recent examples of this process include both insects capable attacking a wide range of host trees (*A. glabripennis* and PSHB), and those infesting a narrow range of hosts (such as *A. planipennis* and *X. glabratus*) (Baranchikov et al., 2008; Fraedrich et al., 2008; Haack et al., 2010; Eskalen et al., 2013; Paap et al., 2018).

Although connections between insect damage and fungal activity in wood were recognized as early as the 19th century (Hartig, 1878), it has been difficult to elucidate the relative roles of each in tree damage as they typically occur in multipartite associations in “noisy” natural systems. However, recent studies have shed light on the potential for pathogens to amplify insect damage (and vice versa), indicating that at least some fungal associates have the ability to catabolize conifer defense compounds and improve beetle tunneling behavior (Wadke et al., 2016; Zhao et al., 2018). Indeed, Zhao et al. (2018) found that bark beetles preferred substrate colonized by fungi and avoided phenolics (plant defense compounds). Although tree damage is often attributed to insects, the role of fungal pathogens in lowering host defenses is likely to be important in many cases: partitioning damage caused by fungi and beetles may be important for targeting mitigating strategies and should be a priority for future research.

Clearly tree resistance is a critical parameter when considering the negative impacts of insect pests and pathogens. Environmental perturbations, especially those associated with climate change, such as droughts, floods, storms, and elevated temperatures, are particularly concerning. These events can cause physical damage to trees (Allen et al., 2010), enhancing the ease of colonization by insects and their accompanying fungal pathogens, while prolonged stress may also impair the ability of trees to direct resources toward defense and repair (Bolton, 2009). For example, inoculation experiments with Norway spruce seedlings have demonstrated that temperature and CO_2_ level increases based on future climate predictions, as well as reduced water availability, can amplify the damage caused by certain fungal species (Linnakoski et al., 2017a,b); although the extent to which these findings are caused by changes in fungal virulence as opposed to host resistance, remains to be determined.

## Concurrent Infections

As part of their lifecycle, beetles can acquire and deposit multiple fungi (both co-evolved symbiotic and opportunistic species) at the same time. For example, of 298 spruce bark beetles sampled during an outbreak in Finland, more than two fungal species were concurrently found on over 40% of individuals (Linnakoski et al., 2016). Indeed, it is very likely that most trees are simultaneously infected by multiple pathogens (co-infections). However, up until recently the prevailing paradigm of infection biology was based on a single pathogen causing a single disease (shown using Koch's postulates), and this out-dated model remains dominant in forest pathology (Tollenaere et al., 2016).

Most research focused on the potential role of co-infections in plant diseases comes from agricultural systems (Lamichhane and Venturi, 2015; Tollenaere et al., 2016). Here studies have demonstrated cases in which severe plant disease can result from co-infections but not from single infections (Rochow and Ross, 1955)—but also that co-infections can reduce the negative impacts of a severe pathogen (Round and Wheeler, 1978). In forestry, a recent study demonstrated the role of co-infections as a cause of Acute Oak Decline (Denman et al., 2018). Fungal viruses (mycoviruses) may also cause changes in fungus aggressiveness (Pearson et al., 2009; Vainio et al., 2018) and therefore alter tree disease outcomes. It is established from forest pathosystems that fungal species and strains interact with each other and differ in their pathogenic potential (Krokene and Solheim, 1998; Repe et al., 2015; Linnakoski et al., 2017a,b). As a result, secondary infections with less virulent species or strains may provide mitigation tools against disease outbreaks.

## Control of Fungal Forest Infections

The most efficient strategy to protect trees is to prevent the introduction of wood-boring insects and their associated pathogens. Unfortunately, current regulatory efforts are inadequate to detect unknown species (Roy et al., 2014), and in several cases, damaging invasive forest pests and pathogens have been novel to science, or poorly understood in their native environment.

What then can be done? As the transfer of fungal pathogens is intimately connected to wood-boring insects, control efforts targeted at insect vectors are likely to be most effective. Innovative biocontrol tools, such as volatile compounds (VOCs), can form part of an integrated strategy. These regulate insect communication and can be utilized as potential repellents against beetles at outbound transport points or increase the efficacy of luring traps (Kandasamy et al., 2016; Hughes et al., 2017). Following prevention, early detection and range minimization are important, and should occur in urban areas that serve as entry points of invasions (Colunga-Garcia et al., 2010; Paap et al., 2017), and by forest monitoring and removal of infested trees by sanitation cuttings. More widespread strategies include the use of other pathogens as biocontrol agents to enhance the natural defense mechanism of trees and interfere with infections (Postma and Goossen-van de Geijn, 2016). For example, entomopathogens can be used as biocontrol agents against wood-boring beetles (Hajek and van Frankenhuyzen, 2017), and mycoviruses can be utilized in the control of fungal forest pathogens (Pearson et al., 2009; Vainio et al., 2018). However, the development of any biological control tool is a long process, which first requires sound knowledge of each organism involved.

## Conclusions

Managing increasingly disturbed forests will be an important global challenge moving forward. While significant research effort has been devoted to understanding and mitigating the effects of insect pests on forest health (Vega and Hofstetter, 2015), in this article we highlight the comparatively neglected role of the fungal pathogens they carry. These are able to inflict significant forest damage (Wingfield et al., 2016), and it is likely that their negative impacts will be amplified over future decades due to environmental perturbations associated with climate change.

Fundamental baseline information on the diversity and frequency of most insect-fungal associations is currently lacking. Such information is important for recognizing novel vector-fungi associations, which have the potential to cause serious forest damage (Ploetz et al., 2013; Wingfield et al., 2016), and may aid in the timeliness of risk assessments and mitigation strategies following distributional changes of insect pests. The frequency and extent to which fungal associates facilitate and amplify insect damage is an intriguing line of enquiry and another area in need of research attention. Although evidence is currently limited to a small number of studies and systems, such as Dutch elm disease (Ploetz et al., 2013; Santini and Faccoli, 2013), it may be a common phenomenon associated with insect damage and provide a potential means to limit their impact.

While important advances have been made in understanding the impacts of fungal pathogens associated with forest insect pests, many pertinent questions remain and we hope this article will help stimulate research to investigate them.

## Author Contributions

All authors listed have made a substantial, direct and intellectual contribution to the work, and approved it for publication.

### Conflict of Interest Statement

The authors declare that the research was conducted in the absence of any commercial or financial relationships that could be construed as a potential conflict of interest.
